# Case Report: Respiratory outcome in a preterm infant following previable rupture of membranes and persistent oligohydramnios

**DOI:** 10.3389/fped.2024.1457129

**Published:** 2025-01-15

**Authors:** Akila Subramanian, Ong Kai Zhi, Arun Kumar Pugalenthi, Victor Samuel Rajadurai, Lay Kok Tan, Suresh Chandran

**Affiliations:** ^1^ Department of Neonatology, KK Women’s and Children’s Hospital, Singapore, Singapore; ^2^ Department of Maternal-Fetal Medicine, KK Women’s and Children’s Hospital, Singapore, Singapore; ^3^ Department of Respiratory Medicine, KK Women’s and Children’s Hospital, Singapore, Singapore; ^4^ Pediatric Academic Clinical Programme, Lee Kong Chian School of Medicine, Singapore, Singapore; ^5^ Pediatric Academic Clinical Programme, Duke-NUS Medical School, Singapore, Singapore; ^6^ Pediatric Academic Clinical Programme, Yong Loo Lin School of Medicine, Singapore, Singapore

**Keywords:** prematurity, oligohydramnios, prolonged rupture of membranes, pulmonary hypoplasia, persistent pulmonary hypertension, pulse oximetry study

## Abstract

Mid-trimester preterm premature rupture of membranes is a rare complication of pregnancy associated with significant maternal and fetal risks. The ensuing prolonged oligohydramnios can lead to fetal pulmonary hypoplasia. In addition, there is an increased risk of miscarriage, preterm birth, and chorioamnionitis, contributing to septic morbidity in the mother–baby dyad. This case report describes the management and outcomes of an infant born at 32 weeks following the rupture of membranes at 16 weeks of gestation, resulting in severe oligohydramnios. Soon after birth, the infant had respiratory compromise, requiring high-frequency oscillatory ventilation and nitric oxide. Despite the initial poor prognosis, the infant remained stable with various ventilation modalities managed by a multidisciplinary team. He was discharged home after 108 days in the hospital and remained on non-invasive ventilatory support until 8 months of age while the home care and hospital specialty teams monitored him. The favorable respiratory outcome of this case is a rarity for cases with similar clinical circumstances, in which the managing team counsels parents about poor fetal outcomes and many proceed to terminate the pregnancies. In this reported case, we highlight the importance of multidisciplinary and interprofessional team management from antepartum monitoring and planning delivery time to subsequent short- and long-term postnatal care involving maternal-fetal medicine specialists, neonatologists, pediatric cardiology and respiratory specialists, and home care teams.

## Introduction

The incidence of mid-trimester preterm premature rupture of membranes (PPROM) is reported to be 0.4%–0.7% of pregnancies (1). This results in the loss of amniotic fluid (AF), and oligohydramnios ensues. In the embryonic period, AF is contributed by the maternal serum, coelomic fluid, and amnion. Later, with renal function beginning at 10 weeks of gestation, amniotic fluid is primarily produced by fetal urine, with contributions from lung secretions (2, 3). Animal studies have shown the need for adequate lung fluid secretion for the optimal growth and development of fetal lungs (4–6). The lung luminal pressure is higher than the AF pressure and the vocal cords act as a one-way valve. The lung fluid is secreted into the lumen against a resistance imparted by the laryngeal abduction, nasopharynx, and chest wall recoil pressure (7–9). This luminal distending pressure is crucial for lung growth.

While there are diverse etiologies for oligohydramnios in the mid-trimester gestation, PPROM poses a significant challenge as prolonged oligohydramnios hinders fetal development. The available evidence suggests that the volume of lung fluid at term equals the functional residual capacity (FRC) of the lungs, which determines the compliance of the lungs, chest wall, and the distending pressure, which are critical factors determining lung growth (10–12). Thus, prolonged leaking of lung fluid hinders the development of liquid FRC, resulting in hypoplastic lungs (4). Pulmonary hypoplasia (PH) presents as a neonatal emergency with severe respiratory compromise resulting from a reduced number of alveoli or bronchopulmonary segments. PH secondary to mid-trimester PPROM has been reported with a high perinatal mortality rate (1). We present a favorable respiratory outcome in an infant born at 32 weeks with maternal oligohydramnios following PPROM from 16 weeks of gestation.

## Case presentation

A primigravida mother was booked for antenatal care at 5 weeks of gestation in our center. Her routine antenatal tests, incorporating fetal aneuploidy screening, including cell-free fetal DNA non-invasive prenatal screening, were normal and reassuring. However, a screening fetal anomaly scan performed at 20 + 3 weeks showed a significantly reduced amniotic fluid index (AFI) of 1.8 cm. Fetal biometry was appropriate for gestational age (GA). The kidneys and bladder were visualized. The mother had experienced leaking liquor from 16 weeks of gestation, and this was confirmed on vaginal speculum examination with a pool of liquor in the vaginal fornix. There was no maternal pyrexia or foul-smelling vaginal discharge.

The examination was unremarkable, with a soft, non-tender abdomen and uterus. An Actin PROM swab test was positive for AF. The couple was counseled on the diagnosis and the poor prognosis by the obstetric team. Fetal medicine specialists and the neonatal team gave a guarded prognosis. The team explained oligohydramnios and related risks of fetal loss, PH, limb contractures, and chorioamnionitis. The couple was also informed of the complications of prematurity and the need for a prolonged Neonatal Intensive Care Unit (NICU) stay if the pregnancy continued past the threshold of neonatal viability. Their options were discussed, including termination of pregnancy, and the couple opted for expectant management.

The mother was initiated on antibiotics and strict bed rest was advised. Blood counts and infective markers were unremarkable. The vaginal swab was positive for *Gardnerella vaginalis*, negative for Group B *Streptococcus*, and the urine culture was sterile.

She was reviewed in the outpatient clinic weekly and advised to get admitted if she developed symptoms or signs of sepsis or preterm labor. Her infective markers were serially monitored, trended, and remained negative. At 24 weeks of gestation, antenatal corticosteroids were given. The AFI remained persistently low, between 1.0 and 1.7 cm, but maintained fetal growth.

At 31 + 4 weeks, the mother complained of abdominal pain and blood-stained discharge. The cervix was closed, and there was no foul-smelling discharge. An ultrasound scan (US) showed the fetus in breech presentation and oligohydramnios. The clinical impression was chorioamnionitis. She was admitted and was given antibiotics. The cardiotocograph showed a reassuring trace. A multidisciplinary team discussion was held to discuss delivery plans, and the team reached a consensus on delivering the baby. She underwent a cesarean section after a second course of corticosteroids and magnesium sulfate at 32 weeks of gestation. The neonatal team was on standby. Placental swabs and histopathology were negative for chorioamnionitis. The mother was discharged home on day 4.

**Figure 1:**
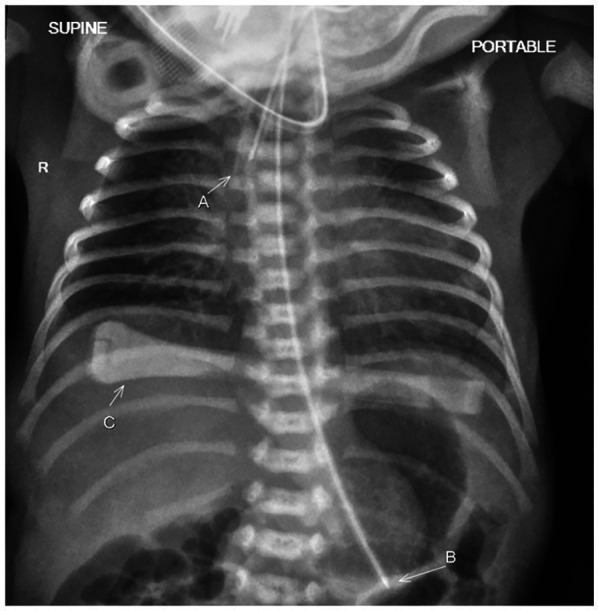
The chest radiograph taken just after birth shows bilateral small radiolucent lungs in keeping with bilateral pulmonary hypoplasia. The patient has been intubated with the endotracheal tube seen in the trachea (A). A feeding tube (B) and an umbilical cord clamp (C) are also present.

A male infant weighing 1,550 g [26th percentile, −0.65 standard deviation scores (SDS)] with a head circumference of 28.5 cm (27th percentile, −0.60 SDS) and a length of 38 cm (6th percentile, −1.58 SDS) was born with an Apgar score of 5 and 9 at 1 and 9 min, respectively. Cord blood gas showed a pH of 7.2 and a base excess of +0.3. The baby had an initial weak cry. He quickly became apneic and was given positive pressure ventilation. He required positive inspiratory pressure/positive end-expiratory pressure (PIP/PEEP) of 25/5 cm H_2_O with a fraction of inspired oxygen (FiO_2_) of 1. With the return of spontaneous respiratory effort at 5 min, he was given continuous positive airway pressure (CPAP, cm H_2_O) support of +5. A chest radiograph showed bilateral small radiolucent lungs (Figure 1). However, oxygen saturations remained in the low 80s, and the baby was intubated at 8 min of life. A blood culture was taken, and antibiotics (penicillin and gentamicin) were initiated. There were no dysmorphic features, limb contractures, or chest deformity.

In the NICU, the baby remained ventilated with two doses of surfactant at 49 min and 11 h of life, respectively, with inadequate response. At 14 h of life, he had oxygenation failure. An echocardiography revealed severe pulmonary hypertension (PHT) and a patent ductus arteriosus (PDA) of 2.6 mm with a right-to-left shunt. The respiratory support was escalated to high-frequency oscillatory ventilation (HFOV) with a graded-up setting to a mean airway pressure of 18, frequency of 12, amplitude of 36, and FiO_2_ of 1. Inhaled nitric oxide (iNO) of 20 ppm was added at 15 h of life. The team counseled the parents regarding the severity of the illness and possible poor outcomes. Blood counts, septic markers, and the blood culture were unremarkable, and antibiotics were discontinued on day 3 of life.

The pre/post-ductal saturations and blood gases improved over 8 h of iNO. By day 8 of life, he was weaned off iNO and converted to a conventional ventilator. On day 15, he was extubated to a non-invasive nasal mask with bilevel positive airway pressure (BiPAP) ventilation using a PIP/PEEP of 22/8 cm H_2_O and FiO_2_ of 0.3. He was then converted to CPAP of +8 on day 17 of life with stable blood gases while on FiO_2_ of 0.3.

On day 50, he had an increasing oxygen requirement; his BiPAP pressures were increased to 22/9 cm H_2_O, respiratory rate (RR) of 40/min, and FiO_2_ to 0.5. His chest x-ray showed increased bilateral diffuse peribronchial airspace opacification and cuffing, suggesting bronchopulmonary dysplasia. Subsequently, he was commenced on oral prednisolone for 14 days as per the Bhandari protocol (13). He responded well; on day 78, he was converted to CPAP + 7 and FiO_2_ of 0.24. The pediatric respiratory team reviewed him. He underwent an overnight pulse oximetry test (POT) on day 87 of life, which revealed suboptimal results (Table 1). A POT was performed using a Masimo Radical-7 oximeter with a signal averaging three seconds. Profox™ software was used to download and analyze the oximetry indices. The oximetry data were analyzed and reported by a pediatric respiratory consultant. The respiratory specialist team recommended investigating gastroesophageal reflux (GER). Medical management of GER was unsuccessful. The pediatric gastrointestinal specialist team advised an upper gastrointestinal contrast study that confirmed moderate GER and a nasojejunal (NJ) tube was inserted. On day 93, a POT was conducted, and the results warranted an escalation of respiratory support to BiPAP. On day 100, the POT showed the percentage of time SpO_2_ was <90% improved from 6.2% to 1.7%.

**Table 1:** Serial pulse oximetry test data during the treatment period.

CA	Day 87 of life	Day 93 of life	Day 100 of life	3 months of age	3.5 months of age	4 months of age	5 months of age	6 months of age	7 months of age	8 months of age
NIV setting	CPAP + 9 O_2_ 2 L/min	CPAP + 9 O_2_ 2 L/min	BiPAP 16/8 O_2_ 2 L/min	BiPAP 16/8 O_2_ 1 L/min	BiPAP 14/8 O_2_ 1.5 L/min	BiPAP 12/7 O_2_ 1 L/min	BiPAP 12/7 O_2_ 1 L/min	CPAP + 7 O_2_ 1 L/min	NC O_2_ 1 L/min	RA
Desat index	48.6	47.4	49.9	37.5	8.3	10.5	6.3	9.1	15.5	11
Mean SpO_2_	94.7%	94.8%	95%	95%	97%	97%	97%	97.6%	98%	96%
Desat nadir	79%	69%	77%	84%	90%	87%	91%	91%	90%	87%
% time SpO_2_ <90%	6.1%	6.2%	1.7%	0.9%	0%	0%	0%	0%	0%	0%

BiPAP; bilevel positive airway pressure; CA, corrected age; CPAP, continuous positive airway pressure; Desat, desaturation; NC, nasal cannula; NIV, non-invasive ventilation; O_2_, oxygen; RA, room air; SpO_2_, oxygen saturation.

Pulse oximetry study reports on days 87, 93, 100, and 3, 3.5, 4, 5, 6, 7, and 8 months of life.

Home care training for the parents was initiated by the neonatal home care team, which included a respiratory therapist, physiotherapist, dietitian, speech and language therapist (SLT), and specialized trained nurses for infant home care. The parents/carers were trained and certified in home non-invasive ventilation machines, tube feeding, vital sign monitoring devices, infant cardio-pulmonary resuscitation, and foreign body airway obstruction management. The parents were educated on red flags related to NJ feeding. The carers were allowed to contact the home care team whenever necessary. Periodic home visits by our nursing team recorded the progress. The baby was discharged home on day 108 of life on continuous NJ tube feeding, home BiPAP machine support of 16/8, RR of 30/min, and an oxygen flow of 2 L/min. The neonatal home care team followed up with the baby. The respiratory and neonatal consultants made weekly phone calls to the parents and conducted fortnightly/monthly clinical reviews. The dietitian and SLT assessed him during joint clinic sessions. He was also enrolled in a preterm neurodevelopmental follow-up program.

A series of POTs (Table 1) performed at home showed improvement in oximetry indices, enabling the gradual weaning of respiratory support to CPAP + 7 by 5 months of age. As the baby remained stable with good oximetry indices at 6 months, we gradually weaned him onto nasal cannula oxygen.

**Figure 2:**
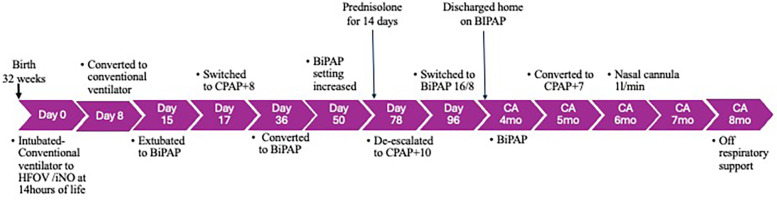
Timeline of events from birth to 8 months of age. BiPAP, bilevel positive airway pressure; CA, corrected age; CPAP, continuous positive airway pressure; HFOV, high-frequency oscillatory ventilation; NIV, non-invasive ventilation.

Subsequently, the nasal cannula oxygen was weaned to room air by a corrected age of 8 months. Following this, his oximetry indices in room air were satisfactory (Figure 2).

A serial cardiac evaluation documented the spontaneous closure of the PDA with no PHT at 2 months of age. He was initiated on palivizumab prophylaxis before discharge. His hearing and retinopathy of prematurity screenings were normal. He had a normal cranial US at birth and 2 and 4 months. His renal and liver function tests from birth remained stable. At 4 months, he was converted to bolus feeding and was bottling by 6 months of age.

He was periodically assessed by neonatal, respiratory, and cardiac specialists; an SLT; and a dietitian at 3–6 weekly intervals. At the corrected age of 14 months, his growth parameters were as follows: weight 9,300 g (100th percentile, +73.77 SDS), head circumference 45 cm (11th percentile, −1.22 SDS), and height 79.3 cm (69th percentile, +0.50 SDS). His periodic neurodevelopmental assessment was conducted using the “Ages and Stages Questionnaires” (ASQ-3 and ASQ: SE-2) appropriate for his age and was within the normal range.

## Discussion

We presented a mother who had PPROM and oligohydramnios from 16 weeks of gestation. The infant was delivered at 32 weeks of gestation after two courses of antenatal steroids. The preterm infant had a turbulent immediate neonatal period. Endotracheal intubation, surfactant administration, ventilation using HFOV/iNO, postnatal steroids, non-invasive ventilation, and home care support resulted in a favorable outcome at 8 months. Sequential POTs monitored his progress in respiratory status.

Oligohydramnios is characterized by an AFI <5 cm or a single deepest pocket (SDP) <2 cm (14). Isolated oligohydramnios occurs without any fetal and maternal underlying conditions, and the incidence is 1%–5% of all pregnancies (15). Pulmonary hypoplasia is evident in 18% of cases with oligohydramnios (14).

The etiology of oligohydramnios can be antenatal factors or fetal abnormalities. Fetal abnormalities resulting in oligohydramnios include chromosomal and renal anomalies, impairing urine production, which is essential for maintaining AF volume (14, 16). Mid-trimester PPROM results in acute and dramatic oligohydramnios. The rupture of membranes can potentially trigger uterine contractions and cervical dilatation, resulting in either miscarriage or preterm birth, and increases the risk of chorioamnionitis, leading to maternal sepsis and perinatal infective morbidity. Rahman and Pervin (17) reported a study in which 5.2% of patients had oligohydramnios, with 66% attributed to PPROM, with unfavorable neonatal outcomes including perinatal death (14%), low birth weight (28%), respiratory depression (32%), and NICU admissions (29%). The First Look Study performed in low- and middle-income countries reported an association of oligohydramnios with higher rates of adverse maternal, fetal, and neonatal outcomes, suggesting the need for effective prediction, diagnosis, and interventions (18). Antenatally, the prediction and diagnosis of PH using fetal lung-to-bodyweight ratio and 2D and 3D USs has been reported by Ruano et al. (19). Messerschmidt et al. (20) reported that lung volumetric assessments using MRI in 16–27-week fetuses can predict neonatal mortality with a sensitivity of 80% and specificity of 86%.

The pathophysiology of PH secondary to oligohydramnios involves disruption of fetal pulmonary development in or before the canalicular stage. The reduced volume of AF can lead to compression of the fetal chest cavity, limiting the expansion of the lungs and impeding their optimal development (1). This compression restricts the growth of lung tissue and interferes with alveolarization, resulting in decreased lung volume and compromised respiratory function. The mechanical constraint on lung development may also lead to PH with fewer, smaller alveoli and reduced airway arborization (16). Consequently, neonates born with PH face significant challenges in gas exchange, often requiring intensive medical intervention to support respiratory function. There is a strong correlation between the duration of oligohydramnios and the severity of PH, with longer durations of PPROM leading to more pronounced lung underdevelopment (21, 22).

The consequences of oligohydramnios extend beyond PH to affect other neonate organ systems. Kim et al. (16) found that PPROM onset <25 weeks gestation with severe oligohydramnios increased the odds of neonatal death, PH, severe PHT, and retinopathy of prematurity (ROP). Other complications may include growth restriction, neonatal sepsis, musculoskeletal contractures, umbilical cord compression resulting in fetal distress, and meconium aspiration syndrome. In addition, severe oligohydramnios may increase the risk of cesarean delivery and NICU admission (23, 24).

McIntosh (25) coined the term dry lung syndrome as a milder end of the oligohydramnios spectrum. He described this as a temporary “functional hypoplasia” due to a leak of the lung fluid and collapse of the airway, necessitating high inflation pressures to reopen them. The other end of the spectrum is PH, a postmortem pathological diagnosis defined by lung DNA/bodyweight ratio and radial alveolar counts. PH occurs with severe deformations, but mild or no deformations are also seen with severe PH. The clinical diagnosis of PH in an infant rests on oligohydramnios, low Apgar scores, deformities, and an unexpected respiratory course (26).

Williams et al. (23) indicated that survival with short-term morbidity among high-risk patients was 73%, with a favorable response to HFOV and iNO therapy, highlighting the reversibility of PHT in infants with PH with iNO therapy. Linehan et al. (27) reported a neonatal survival rate of 20% when PPROM was diagnosed between 14 and 23 + 6 weeks. Winn et al. (24), in a prospective study, found PH in 12.9% of PPROM cases and a perinatal mortality rate of 54%. Logistic regression analysis revealed that the GA at the rupture of the membranes, latency period, and initial or average AFI were critical, independent predictors of PH and the former two were predictors of perinatal death. All three predictors were observed in our case. Blott and Greenough (28) reviewed outcomes of pregnancies complicated by PPROM in the second trimester, reporting a neonatal mortality rate of 36%, mainly attributed to PH.

After parental training, survivors can be discharged home on non-invasive ventilation with home care support. Multidisciplinary care with sequential POTs and echocardiography for PHT can facilitate successful weaning of respiratory support, as noted in our case.

Long-term outcome studies of infants born to mothers with PPROM and oligohydramnios are limited. The 2-year outcome of a cohort with PPROM at <25 weeks gestation and oligohydramnios showed no statistically significant difference in neurodevelopmental outcomes compared to an aged-matched group of infants (29). Conversely, Simons et al. (30) conducted a prospective cohort study of 86 singleton pregnancies with previable PPROM at a median GA of 20 + 2 weeks, and delivery was at a median of 22 + 6 weeks. In total, 52 women (60.5%) delivered at <24 weeks of gestation. The perinatal mortality rate was 63/86 (73.3%). None of the four fetuses <16 weeks survived to discharge. Of the 23/86 (26%) who survived to discharge, long-term follow-up was available for 13/23(56.5%), of whom 50% at 2 years and 57.1% at 5 years were experiencing respiratory issues.

In our case, the respiratory and hemodynamic consequences of PH were severe in the immediate neonatal period. The impaired morphological maturation of the lungs with severe PHT complicated the neonatal transition. A tailored approach to mechanical ventilation using HFOV with iNO helped our patient overcome hypoxemic failure and PHT.

## Strengths and limitations

The comprehensive evaluation process and step-by-step management approach by a multidisciplinary team outlined in this report serve as a valuable reference for practitioners. The vital feature lies in using POTs for the longitudinal evaluation of his respiratory status. The outcome showcases the possibility of achieving a favorable outcome in patients with severe oligohydramnios from a mid-trimester PPROM. However, as a single case report, our findings cannot be generalizable to all patients who present this way.

## Conclusion

Over the years, neonatal mortality following mid-trimester PPROM and oligohydramnios has shown improvement. Factors contributing to this trend include early diagnosis, rigorous antenatal care, fetal monitoring, antenatal steroids, surfactant therapy, HFOV/iNO, postnatal steroids, and non-invasive respiratory support with periodic POTs. This case report highlights the importance of vigilant monitoring and timely intervention by a multidisciplinary team in pregnancies affected by PPROM and oligohydramnios. Early detection and management of PPROM through interventions such as strict bed rest, antibiotics, close fetal surveillance, and antenatal steroids at the limit of viability can help to optimize outcomes.

## Data Availability

The original contributions presented in the study are included in the article/Supplementary Material, further inquiries can be directed to the corresponding authors.
